# A ‘rule of 0.5’ for the metabolite-likeness of approved pharmaceutical drugs

**DOI:** 10.1007/s11306-014-0733-z

**Published:** 2014-09-19

**Authors:** Steve O′Hagan, Neil Swainston, Julia Handl, Douglas B. Kell

**Affiliations:** 1 School of Chemistry, The University of Manchester, 131 Princess St, Manchester, M1 7DN UK; 2 The Manchester Institute of Biotechnology, The University of Manchester, 131 Princess St, Manchester, M1 7DN UK; 3 School of Computer Science, The University of Manchester, 131 Princess St, Manchester, M1 7DN UK; 4 Manchester Business School, The University of Manchester, 131 Princess St, Manchester, M1 7DN UK

**Keywords:** Genome-wide metabolic reconstruction, Recon 2, Cheminformatics, KNIME, Metabolite-likeness, Drug-likeness

## Abstract

We exploit the recent availability of a community reconstruction of the human metabolic network (‘Recon2’) to study how close in structural terms are marketed drugs to the nearest known metabolite(s) that Recon2 contains. While other encodings using different kinds of chemical fingerprints give greater differences, we find using the 166 Public MDL Molecular Access (MACCS) keys that 90 % of marketed drugs have a Tanimoto similarity of more than 0.5 to the (structurally) ‘nearest’ human metabolite. This suggests a ‘rule of 0.5’ mnemonic for assessing the metabolite-like properties that characterise successful, marketed drugs. Multiobjective clustering leads to a similar conclusion, while artificial (synthetic) structures are seen to be less human-metabolite-like. This ‘rule of 0.5’ may have considerable predictive value in chemical biology and drug discovery, and may represent a powerful filter for decision making processes.

**Electronic supplementary material:**

The online version of this article (doi:10.1007/s11306-014-0733-z) contains supplementary material, which is available to authorized users.

## Introduction

The declining productivity of the drug discovery process is well known (e.g. Empfield and Leeson 2010; Hay et al. 2014; Kell 2013; Kola 2008; Kola and Landis 2004; Rafols et al. 2014; van der Greef and McBurney 2005). Thus, many groups have sought to assess in silico those structural or biophysical properties of successful drugs that might be used as filters to enrich the contents of drug discovery libraries with molecules that share those properties. This has therefore led to concepts such as “drug-likeness” (e.g. Empfield and Leeson 2010; Hay et al. 2014; Kell 2013; Kola 2008; Kola and Landis 2004; van der Greef and McBurney 2005), “lead-likeness” (Gozalbes and Pineda-Lucena 2011; Holdgate 2007; Oprea et al. 2007, 2001; Wunberg et al. 2006), and “ligand efficiency” (Hopkins et al. 2014) by which the potentially desirable properties of such molecules have been assessed.

We recognise that any molecule bioactive in human cells (whether as a drug or for purposes of chemical genomics) must cross at least one membrane, that nutrients necessarily do so, that natural products remain a major source of successful (marketed) pharmaceutical drugs (Gozalbes and Pineda-Lucena 2011; Holdgate 2007; Oprea et al. 2007, 2001; van Deursen et al. 2011; Wunberg et al. 2006), and that successful drugs require or at least use membrane transporters (Dobson et al. 2009; Dobson and Kell 2008; Giacomini and Huang 2013; Giacomini et al. 2010; Kell 2013; Kell and Dobson 2009; Kell et al. 2013, 2011; Kell and Goodacre 2014; Lanthaler et al. 2011) that normally are used for the transport of intermediary metabolites (Herrgård et al. 2008; Swainston et al. 2013; Thiele et al. 2013). Given the natural role for these transporters as transporters of intermediary metabolites, we and others have thus suggested (hypothesised) that successful drugs are in fact much more like metabolites (we use this term to mean the natural intermediary metabolites of human metabolism, and do not consider metabolites of the drugs) than are the typical structures found in drug discovery libraries (e.g. Chen et al. 2012; Dobson et al. 2009; Feher and Schmidt 2003; Gupta and Aires-de-Sousa 2007; Hamdalla et al. 2013; Karakoc et al. 2006; Khanna and Ranganathan 2009, 2011; Peironcely et al. 2011; Walters 2012; Zhang et al. 2011), and following the principle of molecular similarity (e.g. Bender and Glen 2004; Eckert and Bajorath 2007; Gasteiger 2003; Maldonado et al. 2006; Oprea 2004; Sheridan et al. 2004) that “metabolite-likeness” is therefore a useful criterion for the design of successful drugs (Dobson et al. 2009). At one level, this may not be seen as surprising given the fact that pharmaceutical drugs typically bind to proteins at sites to which endogenous metabolites normally bind, but the recognition of the importance of metabolite-likeness in drug discovery and chemical genomics remains less than complete.

While a variety of metabolite (pathway) databases exist (Ooi et al. 2010) [e.g. ChEBI (de Matos et al. 2012; Degtyarenko et al. 2009; Hastings et al. 2013), HMDB (Wishart et al. 2013), KEGG (Kanehisa et al. 2012, 2014), MetaCyc (Altman et al. 2013; Caspi et al. 2014; Karp and Caspi 2011) and MetaboLights (Haug et al. 2013)], the recent availability of a highly curated consensus map (Recon2) of the human metabolic network (and thus of intermediary metabolites) (Swainston et al. 2013; Thiele et al. 2013) now provides the most suitable starting point for the comparison of drugs that have been approved/marketed [available from DrugBank (Knox et al. 2011; Law et al. 2014)] and metabolites that are known to be part of the human metabolic network. We choose this latter over say HMDB since the measurable presence of a molecule in a human sample (e.g. Dunn et al. 2014) does not exclude that it has a nutritional, xenobiotic or gut microbial origin, and HMDB does contain many ‘metabolites’ that are not in fact produced via pathways containing proteins encoded by the human genome. Indeed Peironcely et al. (2011) noted, for instance, that the ‘metabolite’ debrisoquine was indeed classified in their scheme as a non-metabolite (and it is indeed a marketed drug).

Thus the primary purpose of this work (in contrast to our earlier work (Dobson et al. 2009) that included multiple metabolite databases that were not constrained as here), is to use the availability of Recon2 to assess precisely how ‘metabolite-like’ known drugs are, partly as an aid to developing metrics for determining whether drugs are likely to be substrates for relevant transporters and thus whether they are likely to be bioactive. The availability of Recon2 also allows us to reason sensibly about the nature and extent of metabolite space and how it differs from the kinds of molecules typically found in drug discovery libraries.

## Methods

### Construction of datasets

The list of FDA-approved small molecule drugs was downloaded from DrugBank 3.0 (http://www.drugbank.ca/downloads) in November 2013 as an SDF file and consists of 1491 molecules. This is significantly smaller than the fuller list (7330 ‘drugs’ via Drugbank and KEGGDrug) used previously (Dobson et al. 2009). The list of intermediary metabolites was extracted from the latest version of the Recon2 human metabolic network (Thiele et al. 2013). A further manual curation removed from the ‘drugs’ list (i) ‘drugs’ (mainly nutritional supplements) that are also intermediary metabolites produced by enzymes encoded by the genome and thus part of Recon 2 (though adrenaline was treated as a drug), and (ii) those ‘metabolites’ listed in Recon2 that are xenobiotic in nature or simply metals or salts. However, vitamins and essential amino acids and fatty acids, while not encoded by the human genome, were retained as ‘metabolites’ as they are both necessary for human metabolism and form part of the formal human metabolic network. The resultant data are in Supplementary information S3, and consist of 1113 ‘metabolites’ [cf. 5333 ‘metabolites’ previously (Dobson et al. 2009)] and 1381 ‘drugs’. In addition, data on antimalarial compounds were downloaded from the databases at the EBI (https://www.ebi.ac.uk/chemblntd).

### Software

For the cheminformatics analyses we used the KoNstanz Information MinEr (KNIME, www.knime.org) (Beisken et al. 2013; Berthold et al. 2007; Mazanetz et al. 2012; Meinl et al. 2012; Stöter et al. 2013; Warr 2012). KNIME is a workflow environment somewhat similar to Taverna [with which we have previous experience in systems biology analyses (Li et al. 2008a, b)], but which is slightly more focussed on cheminformatics. The workflows we used here included nodes that made use of libAnnotationSBML (Swainston and Mendes 2009), the Chemistry Development Kit (Beisken et al. 2013; Steinbeck et al. 2003) and the RDKit (Riniker and Landrum 2013a; b; Saubern et al. 2011) (www.rdkit.org/). We also used the software MOCK (Handl and Knowles 2007) for multiobjective clustering.

## Results

### Comparison of Tanimoto distances between drugs and natural metabolites

Our first task was to assess the average chemical (structure) distances between molecules according to a suitable metric. Many molecular descriptors exist for encoding molecules in a manner that allows this (e.g. Bender 2010; Duan et al. 2010; Koutsoukas et al. 2013; Sastry et al. 2010; Sheridan and Kearsley 2002; Todeschini and Consonni 2000; Wang and Bajorath 2010), most commonly referred to as fingerprints (e.g. Faulon and Bender 2010; Flower 1998) and sometimes with rather different properties and outcomes when matched against structures or biological activities (e.g. Dhanda et al. 2013; Medina-Franco and Maggiora 2014). Thus, and while some experience shows that they are not greatly different from each other when simply comparing chemical or structural similarity (Dobson et al. 2009; Riniker and Landrum 2013a), which is the focus of the present paper, we looked at a number of methods for producing molecular fingerprints. Probably most common are fingerprints derived from structural keys such as the 166 Public MDL (Molecular ACCess System) MACCS keys (Durant et al. 2002) based on a predefined dictionary of 166 substructures [that contain most of the important features of a larger 960-key set (McGregor and Pallai 1997)] and hashed to give 1,024 bits.

Given the molecular fingerprint method chosen, there is a more general acceptance of the metrics for the similarity of molecules whose (sub)structures are so encoded; although it has a size-dependence (that does not matter for this analysis), the Tanimoto distance, that effectively encodes the numbers of matching and non-matching substructures, is both easy to calculate and pre-eminent (Maggiora et al. 2014; Willett 2006).

We recognise that some 20 % of recent new chemical entities are prodrugs (15 % in the top 100 drugs) (Huttunen et al. 2011), and that some of these are converted non-enzymically to the active substances; however, these normally do not differ greatly in structural terms from the active substance in the marketed entities, so for convenience we shall use the latter. In contrast to Peironcely et al. (2011), who used supervised learning methods such as random forests [which are very powerful (Knight et al. 2009)] to predict whether a substance was or was not a metabolite, we are here interested only in the structural similarities between candidate molecules and Recon2 metabolites, and we confine ourselves strictly to unsupervised methods of analysis.

**Fig. 1:**
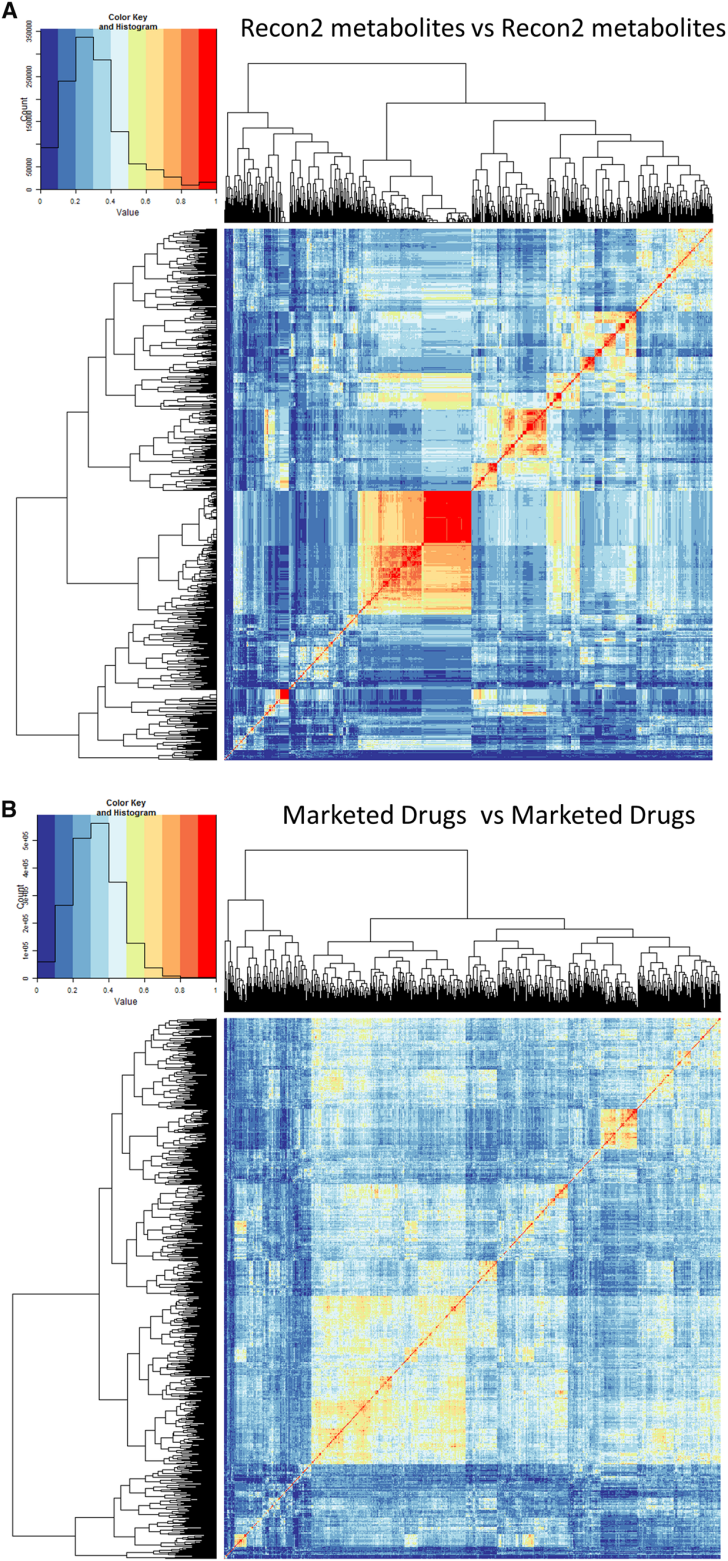

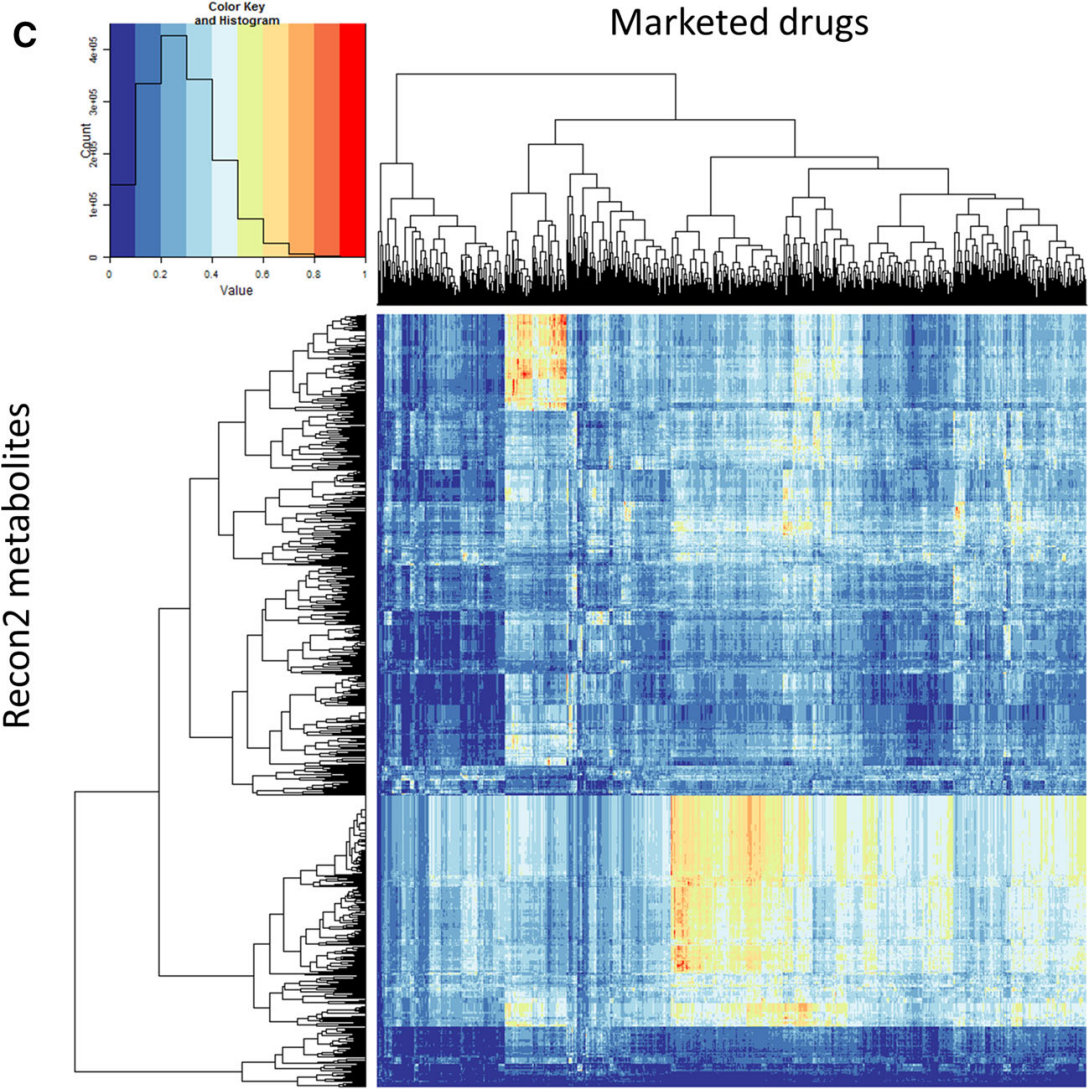
Heat maps of the overall similarities between **a** Recon2 metabolites, **b** drugs and **c** each other. In the latter plot, the drugs lie on the *X*-axis and the metabolites on the *Y*-axis. Chemical structures were encoded using the MACCS encoding and Tanimoto distances calculated as described in Methods. The heat map representation (Eisen et al. 1998) encodes the numbers as a colour; in the present version, for ease of observation, we use ten discrete colours for the ten decades of Tanimoto similarity, with the colours chosen following the recommendations of Brewer et al. (1997) (see also http://www.colorbrewer2.org/). Also shown are hierarchical clusterings of the rows and columns (Eisen et al. 1998) using complete linkage and the default settings in the hclust function in R (Color figure online)

We checked a variety of implementations of the MACCS fingerprints (specifically those used in Open Babel, CDK and RDKit) and found very little difference between them, and for what is presented here we used those in the RDKit implementation. We therefore compared all metabolites against all metabolites (Fig. 1a), all drugs against all drugs (Fig. 1b), and all drugs against all metabolites (Fig. 1c). The metabolite-metabolite similarities (Fig. 1a) reveal multiple clusters, including one that is made up of CoA derivatives (full details in Figure S1), while the clusters of drug-drug similarities Fig. 1b are rather more heterogeneous (the trees are much ‘bushier’). From Fig. 1c, the drug-metabolite similarities, there are some interesting clusters, e.g. the block of red and yellow towards the upper left represented sterols and steroids, while the larger swathe of red and yellow towards the bottom represents mainly CoA derivatives. All the data are given in an addressable form as Excel spreadsheets in Supplementary Information S1–S3.

A number of different fingerprints were used to determine if the extent of closeness of a drug to its nearest metabolite depended greatly on the fingerprint used. The various fingerprints used (http://www.rdkit.org/RDKit_Docs.current.pdf) were provided in the RDKit module (Riniker and Landrum 2013a) (https://code.google.com/p/rdkit/wiki/FingerprintsInTheRDKit) of KNIME (http://tech.knime.org/community/rdkit), and as stated in (Riniker and Landrum 2013b) were atom pairs (AP), feature-based circular fingerprint with radius 2 as bit vector (FeatMorgan2), and a circular fingerprint with radius 2 as bit vector (Morgan2). Morgan2 is the RDKit implementation of the familiar ECFP4, and FeatMorgan2 is equivalent to FCFP4 (Landrum et al. 2011). The features used by the RDKit for FeatMorgan2 consist of various donors, acceptors, aromatic atoms, halogens, basic and acidic atoms. We also used a representation (referred to in KNIME and here as ‘RDKit’) that is said to be a ‘Daylightlike’ topological fingerprint based on hashing molecular subgraphs. Most recently, RDKit has added some extra fingerprints, and for completeness we included these too. Thus, ‘layered’ is an experimental substructure fingerprint using hashed molecular subgraphs, while ‘torsion’ is said to be the bit vector topological-torsion fingerprint for a molecule. As indicated above, all of the data are tabulated in Fig S3.

**Fig. 2:**
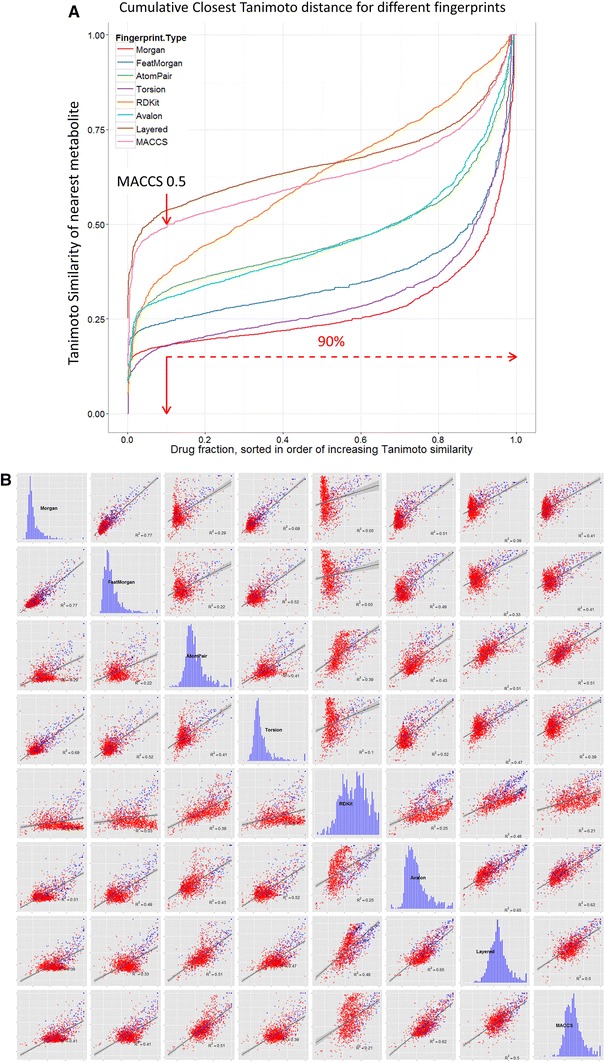

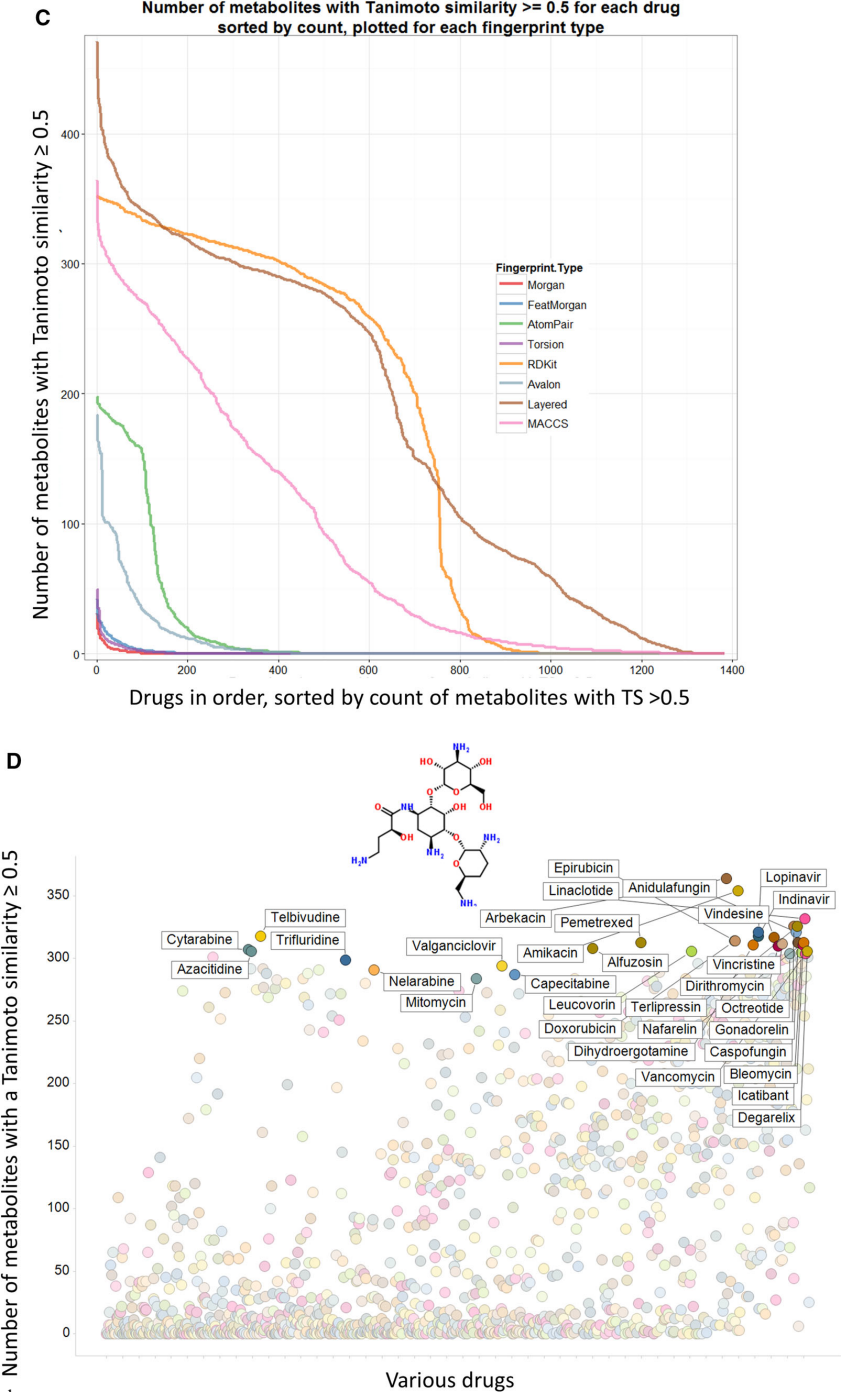

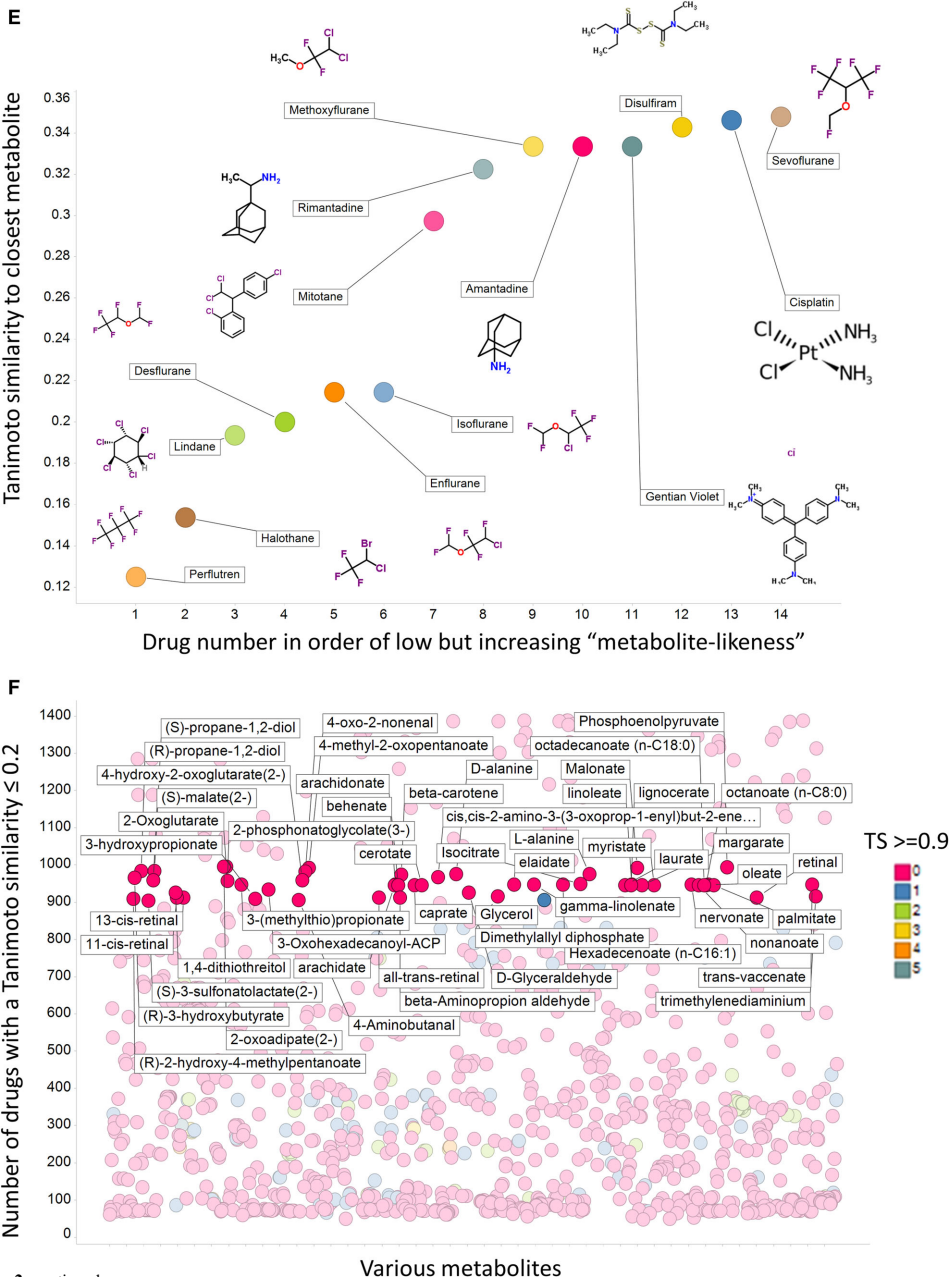
Different structural encodings produce different drug-metabolite distances. **a** Cumulative plots of nearest drug-metabolite Tanimoto distances using various fingerprints. The number of drugs with a Tanimoto similarity of 0.5 or smaller is *arrowed* (i.e. all of those to the right, ca 90 %) have a Tanimoto similarity greater than 0.5. **b** Scatter plots relating the nearest Tanimoto distance to a metabolite for each drug; when the closest metabolites are the same for both encodings they are coloured *red*. Correlation coefficients are as given. The *blue* histograms represent the distributions of Tanimoto similarities for each of the encodings (scaled to fit the relevant windows). **c** Cumulative numbers of metabolites with a Tanimoto similarity ≥0.5 for various drugs and encodings. **d** The variation of the numbers of metabolites with a Tanimoto similarity ≥0.5 for all drugs using the MACCS encoding, with some of the highest labelled by name and with the chemical structure of arbekacin, the ‘most promiscuously metabolite-like’ of all, shown. **e** The 14 least metabolite-like drugs when using the MACCS encoding. **f** An assessment of part of drug-metabolite space where drugs are largely but not entirely distant from metabolites (Color figure online)

Considering first just the Tanimoto similarity (TS) values using MACCS fingerprints and the 1,024 bitstring encoding, 90 % of marketed drugs have a ‘nearest metabolite Tanimoto similarity’ (NMTS, i.e. the TS to the nearest metabolite) of more than 0.5, 98.5 % over 0.4 and 99 % over 0.34, all highly significant values (Baldi and Nasr 2010). The first of those percentages compares with just 12 % when we did not use the ‘genuine’ human metabolites of Recon2 (Dobson et al. 2009) (note that there we used the nearest Tanimoto distance (=1 − TS)). Provided the molecule is not excessively halogenated, its NMTS is over 0.5 (e.g. 0.54 for Chlorzoxazone, 0.55 chlormerodrin, 0.6 diclofenac, 0.65 chlorphenesin and so on). This ‘rule’, by which the very great majority (90 % of) drugs are within a Tanimoto distance of 0.5 in MACCS fingerprint space, may be viewed in the context of the well-known ‘rule of 5’ (Lipinski et al. 1997) (Ro5) mnemonic for predicting drug lead quality. However, the cumulative plots of the NMTS for each drug using different fingerprints (Fig. 2a) do differ quite significantly depending on which fingerprint is used, and clearly the well-established MACCS fingerprints lead to a substantially greater degree of ‘metabolite-likeness’ than do almost all the other encodings (we do not pursue this here). Figure 2 also permits one to read off other metrics such as to note that more than 50 % of drugs have a TS greater than 0.6 to a metabolite for both MACCS and RDKit encodings.

Another indication of the rather different nature of the fingerprints comes from an analysis (Table 1) of the nature, and frequency of occurrence, of the nearest metabolite, where each fingerprint encoding has its own predilections for particular classes of metabolite, reflected also in the overall number of metabolites that are closest to at least one drug. These represent about one quarter of all drugs (or metabolites), an indication of the significant heterogeneity (Hopkins et al. 2014; Paolini et al. 2006) of drug space. RDKit has a slightly unusual predilection for cob(1)alamin and for protoheme, returned as the closest hits on 650 and 73 occasions, respectively (although removing these has negligible effects on the shape of the plot in Fig. 2a, indicating that this lower degree of metabolite-likeness, which is a continuous function, is inherent to the encoding). Scatter plots indicating correlations of ‘nearest metabolites’ with the different encodings are given in Fig. 2b, again illustrating the substantial differences found using the different encodings. Thus we would stress not only that similarity measures differ significantly for the different encodings, but that in functional terms the well-known existence of activity cliffs (e.g. Maggiora et al. 2014) means that quite small differences in molecular similarity may be highly significant with regard to pharmacological effects. In contrast to studies of related molecules that look at this (e.g. Muchmore et al. 2008; Papadatos et al. 2010), we discuss only the similarities themselves.

**Table 1:** Summary of the most frequently represented ‘closest metabolite’ to FDA-approved drugs, the number of times they appear, and the number of metabolites that are closest to a drug at least once

Fingerprint encoding	Most common ‘closest metabolite’	Times represented	Total number of different metabolites that are ‘closest’ to a marketed drug at least once
MACCS	Docosa-4,7,10,13,16-pentaenoic acid	52	359
Atom pair	Linoleic coenzyme A	68	346
Feats Morgan	Docosa-4,7,10,13,16-pentaenoic acid	124	319
Morgan	Docosa-4,7,10,13,16-pentaenoic acid	77	338
RDKit	Methylcobalamin	650	268
Layered	Adenosylcobalamin	87	300
Avalon	Cortisol	65	213
Torsion	Vaccenyl coenzyme A	44	327

In a similar vein, the different encodings produce quite different assessments of the number of metabolites to which each drug displays a Tanimoto similarity exceeding 0.5 (Fig. 2c), with (unsurprisingly, given the data in Fig. 2a) the MACCS, RDKit and Layered encodings showing the greatest tendency towards ‘metabolite-likeness’. Based on MACCS, 50 % of marketed drugs have at least 31 metabolites with a TS of 0.5 or more. The ‘winner’ (i.e. the drug with the most metabolites to which it bears a TS greater than or equal to 0.5) is arbekacin, with 364, and the relevant data, plus a few named drugs, are given in Fig. 2d. It is probably worth commenting, albeit this is not necessarily a surprising finding, that these ‘highly metabolite-like’ drugs are natural products or molecules derived therefrom [see also (Kell 2013; Newman and Cragg 2012)]. The average greatest TS to a metabolite of the five most drug-like drugs (0.547), the five least drug-like drugs (0.683), the five most drug-like Ro5 failures (0.496) and the five least drug-like Ro5 passes (0.557, but minus tegaserod, not present in our list) as listed by Bickerton et al. (2012) are as noted.

By contrast, the substance with the lowest NMTS (perflutren, 0.125) is in fact an injectable contrast agent of lipid microspheres marketed precisely because it does not enter cells, while the next three lowest (NTS ≤ 0.2) are halothane (an inhalational narcotic), lindane (a topical chlorinated insecticide) and desflurane (a polyfluorinated inhalational anaesthetic), consistent with the fact that virtually no natural human metabolites are halogenated. Ten of the 14 least metabolite-like drugs contain at least two halogens (Fig. 2e).

In a similar vein, it is possible to enquire as to which metabolites have the most or fewest marketed drugs closely associated with them in terms of Tanimoto similarity, the latter in particular as a possible indication of areas of chemical space that might be deemed to be relatively underexplored. The metabolites with the very lowest TS to drugs are small and uninteresting (ammonia, water, etc.), so Fig. 2f illustrates those metabolites that are least similar to numbers of drugs between 900 and 1,000, at the same time illustrating the nonlinearity of drug and metabolite spaces by encoding with colours those metabolites that nonetheless have 1–5 drugs with a TS greater than or equal to 0.9 (glycerol is marked and has one, viz. mannitol). One might consider the sparsely populated areas of ‘metabolite-likeness space’ to be ones worth pursuing in drug discovery.

**Fig. 3:**
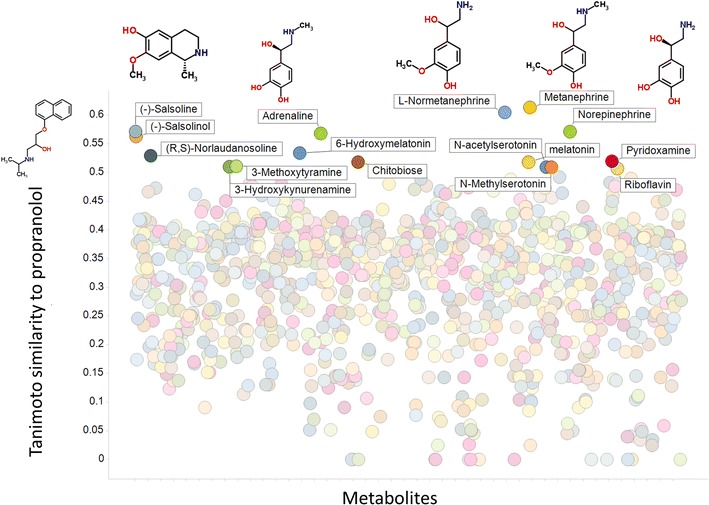
Variation of the Tanimoto similarity for a marketed drug, propranolol, with various metabolites, those with a TS of over 0.5 being labelled, and structures given for a representative set to illustrate the close chemical similarity (Color figure online)

Another means of displaying the data, and a convenient means of interrogating them for a drug of interest, is given in Fig. 3, where we display the Tanimoto similarity to all metabolites for the beta-(adrenergic receptor) blocker propranolol. All metabolites with a TS greater than 0.5 are labelled, and structures are shown for (from left to right) propranolol itself, (−)-salsoline, adrenaline, l-normetanephrine, metanephrine and norepinephrine. While ‘structural similarity’ may be seen as a subjective matter, in this case the chemical similarities are obvious, and it is probably not surprising that a beta-adrenergic antagonist should have similarities of this type.

### Multiobjective clustering of drugs and metabolites

**Fig. 4:**
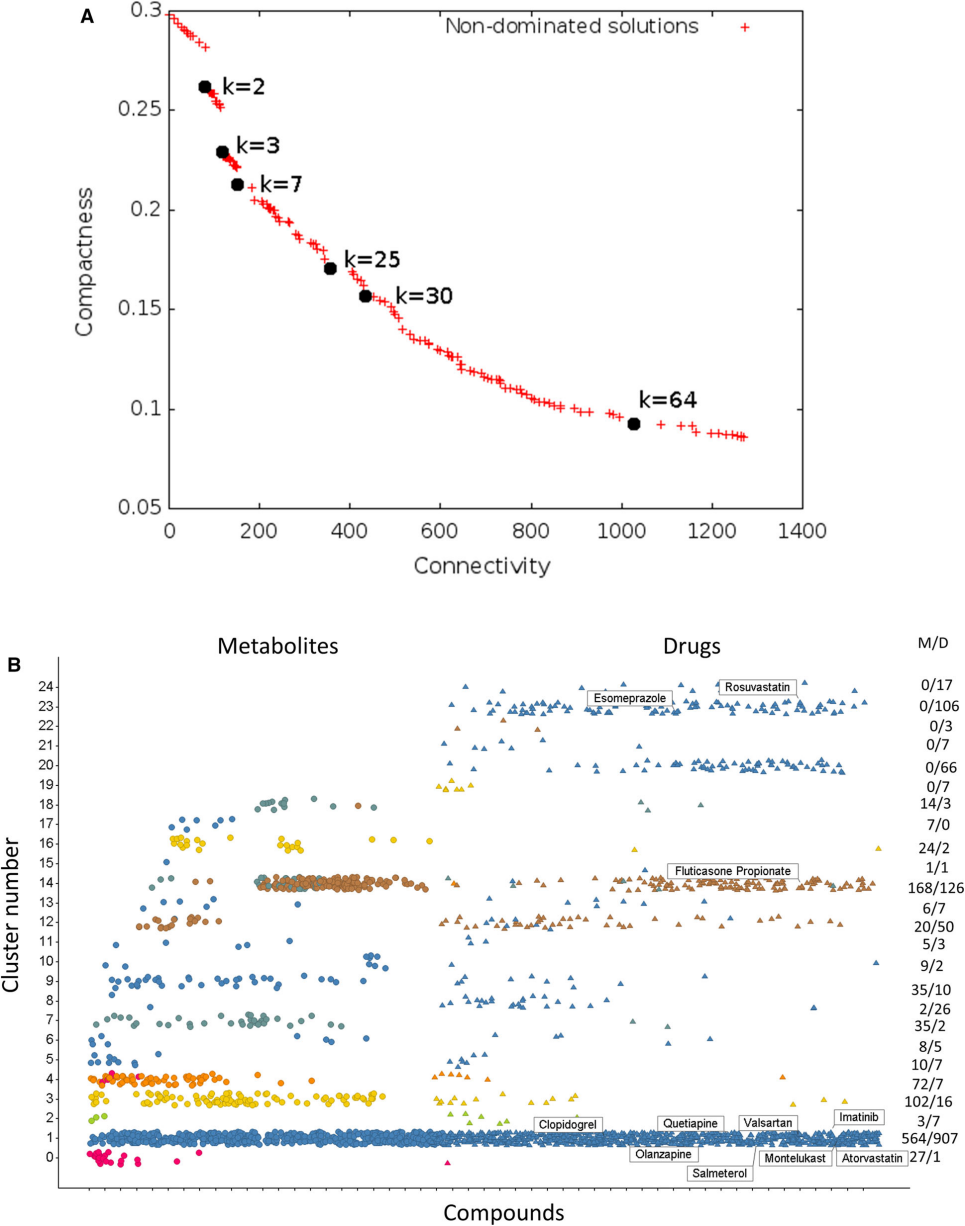
Drug-metabolite clustering using the MACCS encoding and MOCK, a multiobjective clustering algorithm. **a** Dependence of cluster numbers as the weightings of the two main objectives are varied. The ‘knees’ at cluster numbers of 2, 3, 7, 25, 30 and 64 are marked. **b** Cluster membership and its distribution between drugs and metabolites for when 25 clusters are chosen. Data are ‘jittered’ in the *Y* direction to make them clearer (Color figure online)

In the above, we clustered (or bi-clustered) the drugs and the metabolites separately. Another approach to assessing the mapping of drug and metabolite spaces, and the extent to which they overlap or otherwise), is to use clustering methods of both together. These algorithms differ widely [there is no single ‘correct’ clustering (Everitt 1993)] but the state of the art is represented by methods such as MOCK (Handl and Knowles 2007) (MultiObjective Clustering with automatic K) that use multiple objectives [specifically both closeness and connectivity (Handl and Knowles 2007; Handl et al. 2005)] simultaneously to cluster objects on the basis of their ‘similarity’. As with any multiobjective method, there are multiple ‘best’ solutions represented by a Pareto front (Kell 2012), and we illustrate this in Fig. 4. Figure 4a shows the overall variation of ‘optimal’ cluster number for the Pareto front, with ‘knees’ at e.g. 3, 7, 25, 30, 42 and 64 clusters, while Fig. 4b shows the distribution of drugs and metabolites in the MOCK solution for 25 clusters. Also marked are the ‘top ten’ blockbuster drugs by sales from 2010 [NB fluticasone propionate and salmeterol are part of a combined medicine; see also (Kell et al. 2013)], while the colour encodes the cluster membership of compounds when there are only seven clusters. Cluster 0 is mainly small metabolites like bicarbonate, but it is evident that the lower clusters all contain both metabolites and drugs. We also looked at the distribution of various molecular properties (such as polar surface area, molecular mass, log P etc.) between clusters, but no trends nor hotspots were apparent for particular clusters (not shown).

### The drug-likeness of synthetic ‘druglike’ molecules and ‘fragments’ and of natural products

**Fig. 5:**
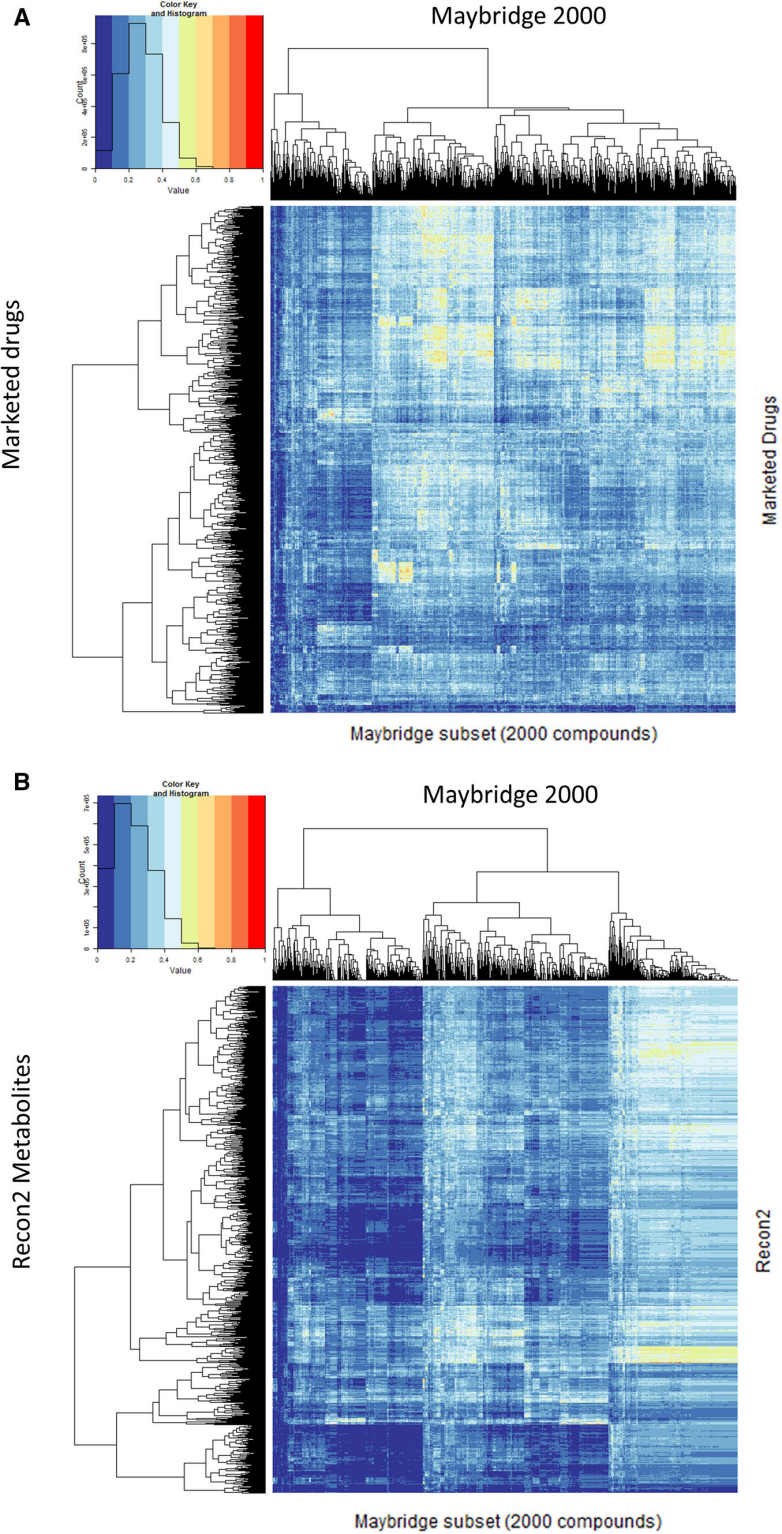

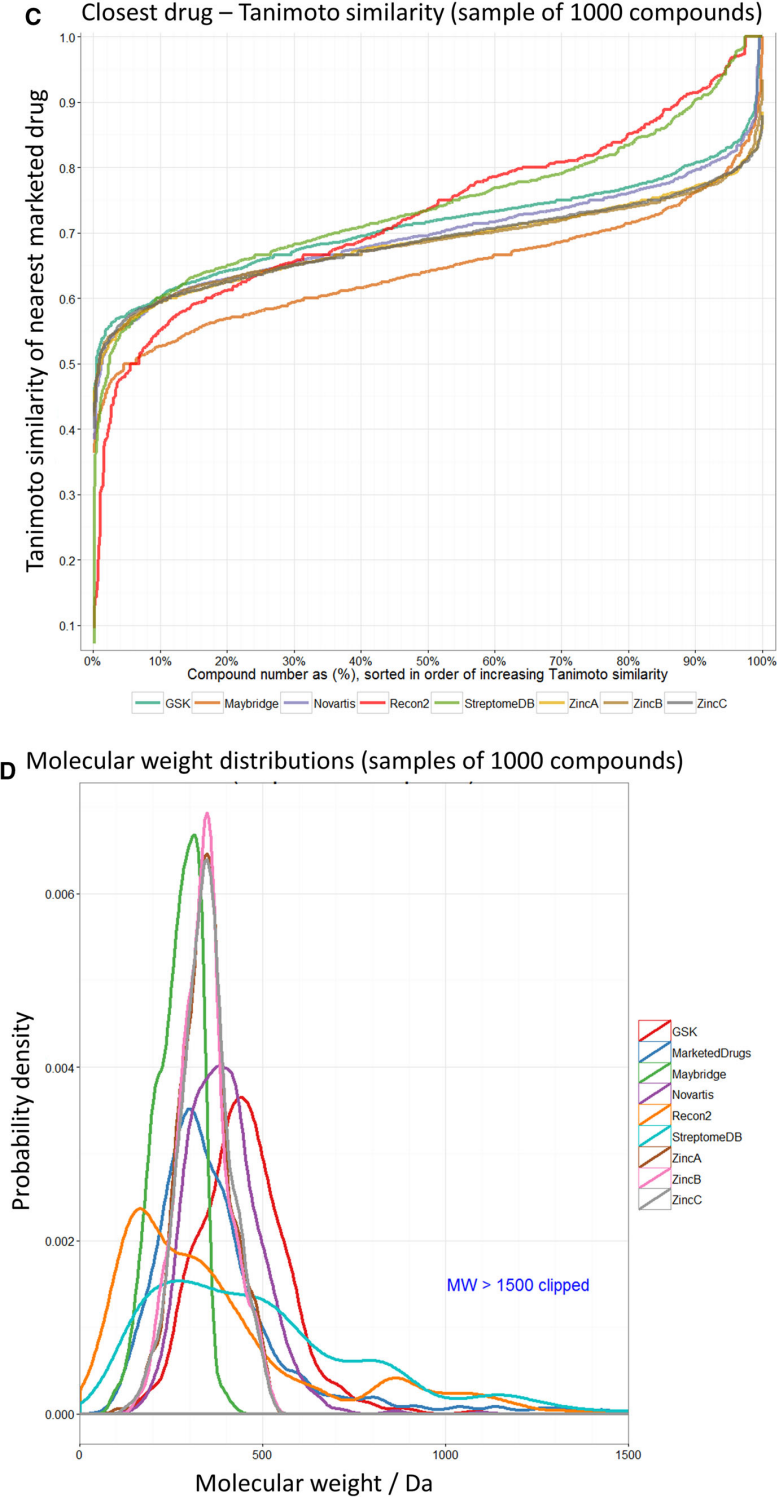
Properties of drugs and drug fragments. **a** Heat map illustrating marketed drug-compound distances of 2,000 drug fragments selected randomly from a Maybridge library (the plot looks very similar for 15,000 fragments). **b** Heat map illustrating metabolite-compound distances of 2,000 drug fragments selected randomly from a Maybridge library (the plot looks very similar for 15,000 fragments). **c** Cumulative plots of nearest marketed drug-compound or marketed drug–fragment Tanimoto distances for various libraries. **d** Distribution of molecular weights for the various datasets used (Color figure online)

Having seen the closeness of successful, marketed drugs to metabolites when both are MACCS-encoded, it was important to establish that (while unlikely) this was not a strange artefact of the MACCS encoding itself. To this end, and while we and others (e.g. Dobson et al. 2009; Feher and Schmidt 2003; Khanna and Ranganathan 2011; Medina-Franco and Maggiora 2014; Ohno et al. 2010) have recognised that marketed drugs do differ structurally from most molecules in drug discovery libraries, despite their ‘biogenic bias’ (Hert et al. 2009), we sought to see how similar such non-marketed drug molecules or compounds are to marketed drugs when we compare them in the same way. The comparison is not entirely favourable to metabolites since we already know (Fig. 2) that many of the very smallest metabolite molecules are simply not druglike, and this is reflected in the data of Fig. 5. Figure 5a shows a heat map relating 2,000 structures taken randomly from the 30,000 in the Maybridge fragment library (similar kinds of map were obtained using subsets of varying sizes up to 15,000) relative to marketed drugs, while Fig. 5b shows that of a random subset of the Maybridge library vs Recon2 metabolites. Figure 5c shows the cumulative similarities (all using MACCS encodings) to metabolites for a collection of molecules from a subset of 1,000 molecules from the Maybridge fragment library, from the 13,533 compounds in the Tres Cantos Antimalarial Drug Set (Gamo et al. 2010), from the 5,697 compounds in the Novartis antimalarial collection (Guiguemde et al. 2010) (note that these last two are in fact ‘hits’ or actives), for 3 subsets of 1,000 molecules from ZINC (Irwin et al. 2012), and of 1,000 from the ~2,400 natural products molecules in StreptomedB (Lucas et al. 2013). We also checked to ensure that we are not biased systematically towards an appearance of metabolite-likeness by say differences in distributions of molecular weights in the different sets, and Fig. 5d shows that we are not, in that a propensity to metabolite-likeness does not seem to follow systematically the MW distribution of the libraries. It is interesting to note that the Novartis and GSK compounds, selected from a very much larger set on the basis of their bioactivity, were even slightly more ‘drug-like’ than were those from Recon 2 at the left-hand end, though Recon 2 was most drug-like overall (note how it and the streptomycete secondary metabolites ‘pull away’ from the other curves beyond 50 %, Fig. 5c), and it seems that no such ‘MACCS artefact’ contributes to the ‘rule of 0.5’. Interestingly, Maybridge tends to contain a rather greater diversity of structures relative to human metabolites, but it is possible that the libraries might be enriched further for possible drugs if they were to include a greater degree of metabolite-likeness. It will obviously be of future interest to determine which fragments or compounds are enriched in molecules that happen to possess particular bioactivities.

## Discussion and conclusions

While both drug and drug target spaces are evidently very heterogeneous (e.g. Adams et al. 2009; Hopkins et al. 2014; Medina-Franco and Maggiora 2014; Paolini et al. 2006), and that is reflected in the analyses presented here, it is highly desirable to be able to find properties that are well represented in marketed (and hence effective and successful) drugs. Given the complexity of drug space, finding a simple mnemonic or rule that has utility is to be welcomed. Indeed, the original ‘rule of 5’ paper states (Lipinski et al. 1997) “This analysis led to a simple mnemonic which we called the ‘rule of 5’ because the cutoffs for each of the four parameters were all close to 5 or a multiple of 5….The ‘rule of 5’ states that: poor absorption or permeation are more likely when: there are more than 5 H-bond donors (expressed as the sum of OHs and NHs); The MWT is over 500; the Log P is over 5 (or M Log P is over 4.15); there are more than 10 H-bond acceptors (expressed as the sum of Ns and Os); compound classes that are substrates for biological transporters are exceptions to the rule.” This famous ‘rule of 5’ (Lipinski et al. 1997) has been highly influential in this regard, but only about 50 % of orally administered new chemical entities actually obey it (Overington et al. 2006; Zhang and Wilkinson 2007) (and see Hopkins et al. 2014); indeed half of recent ‘new chemical entities’ are natural products (Newman and Cragg 2012), that do not obey the Ro5 either. The (also very effective) ‘rule of three’ (Congreve et al. 2003) applies solely to leads and not drugs. While improving drug effectiveness is probably best addressed using combinations of molecules (e.g. Small et al. 2011), we have shown that when encoded using the public MDL MACCS keys, more than 90 % of individual marketed drugs obey a ‘rule of 0.5’ mnemonic, elaborated here, to the effect that a successful drug is likely to lie within a Tanimoto distance of 0.5 of a known human metabolite. While this does not mean, of course, that a molecule obeying the rule is likely to become a marketed drug for humans, it does mean that a molecule that fails to obey the rule is statistically most unlikely to do so. We note that this highlighting of the utility of ‘metabolite-likeness’ as a concept in drug discovery in systems pharmacology is just a first step, as the availability of Recon2 for such analyses open up many new avenues that we do not discuss here. The present analysis has necessarily been retrospective, as we have applied it to existing and successful (i.e. presently marketed) drugs. However, we consider that this rule, and the concept of the utility of metabolite-likeness more generally, may well have significant prospective value in reversing a current trend in medicinal chemistry (Chen et al. 2012; Walters et al. 2011) that runs in a direction precisely opposite to that of metabolite-likeness.

## Electronic supplementary material

Below is the link to the electronic supplementary material.

Table of Recon2–Recon2 similarities, clustered and encoded in an Excel sheet as per the heatmap in Fig. 1a (Tanimoto coefficients are independent of which is the query, so the sheet is symmetrical). Supplementary material 1 (XLSM 10472 kb).

Table of Drug–Drug similarities, clustered and encoded in an Excel sheet as per the heatmap in Fig. 1a. Supplementary material 2 (XLSM 18185 kb).

Table of Recon2–Drug similarities, clustered and encoded in an Excel sheet as per the heatmap in Fig. 1b. RD-HeatMap_all.xlsm.zip. This gives all encodings; the tab using MACCS encodings is that displayed in Fig. 1c. Supplementary material 3 (XLSM 120987 kb).

## References

[CR1] Adams JC (2009). A mapping of drug space from the viewpoint of small molecule metabolism. PLoS Computational Biology.

[CR2] Altman T, Travers M, Kothari A, Caspi R, Karp PD (2013). A systematic comparison of the MetaCyc and KEGG pathway databases. BMC Bioinformatics.

[CR3] Baldi P, Nasr R (2010). When is chemical similarity significant? The statistical distribution of chemical similarity scores and its extreme values. Journal of Chemical Information and Modeling.

[CR4] Beisken S, Meinl T, Wiswedel B, de Figueiredo LF, Berthold M, Steinbeck C (2013). KNIME-CDK: Workflow-driven cheminformatics. BMC Bioinformatics.

[CR5] Bender A (2010). How similar are those molecules after all? Use two descriptors and you will have three different answers. Expert Opinion on Drug Discovery.

[CR6] Bender A, Glen RC (2004). Molecular similarity: A key technique in molecular informatics. Organic & Biomolecular Chemistry.

[CR7] Berthold MR, Preisach C, Burkhardt H, Schmidt-Thieme L, Decker R (2007). The Konstanz Information Miner. Studies in classification, data analysis, and knowledge organization (GfKL 2007).

[CR8] Bickerton GR, Paolini GV, Besnard J, Muresan S, Hopkins AL (2012). Quantifying the chemical beauty of drugs. Nature Chemistry.

[CR9] Brewer CA, MacEachren AM, Pickle LW, Herrmann D (1997). Mapping mortality: Evaluating color schemes for choropleth maps. Annals of the Association of American Geographers.

[CR10] Caspi R (2014). The MetaCyc database of metabolic pathways and enzymes and the BioCyc collection of Pathway/Genome Databases. Nucleic Acids Research.

[CR11] Chen HM, Engkvist O, Blomberg N, Li J (2012). A comparative analysis of the molecular topologies for drugs, clinical candidates, natural products, human metabolites and general bioactive compounds. Medchemcomm.

[CR12] Congreve M, Carr R, Murray C, Jhoti H (2003). A rule of three for fragment-based lead discovery?. Drug Discovery Today.

[CR13] de Matos P, Adams N, Hastings J, Moreno P, Steinbeck C (2012). A database for chemical proteomics: ChEBI. Methods in Molecular Biology.

[CR14] Degtyarenko, K., Hastings, J., de Matos, P., Ennis, M. (2009). ChEBI: An open bioinformatics and cheminformatics resource. *Current Protocols in Bioinformatics*. Chapter 14, Unit 14–9. 10.1002/0471250953.bi1409s26 19496059

[CR15] Dhanda SK, Singla D, Mondal AK, Raghava GPS (2013). DrugMint: A webserver for predicting and designing of drug-like molecules. Biology Direct.

[CR16] Dobson PD, Kell DB (2008). Carrier-mediated cellular uptake of pharmaceutical drugs: An exception or the rule?. Nature Reviews Drug Discovery.

[CR17] Dobson P, Lanthaler K, Oliver SG, Kell DB (2009). Implications of the dominant role of cellular transporters in drug uptake. Current Topics in Medicinal Chemistry.

[CR18] Dobson PD, Patel Y, Kell DB (2009). “Metabolite-likeness” as a criterion in the design and selection of pharmaceutical drug libraries. Drug Discovery Today.

[CR19] Duan J, Dixon SL, Lowrie JF, Sherman W (2010). Analysis and comparison of 2D fingerprints: Insights into database screening performance using eight fingerprint methods. Journal of Molecular Graphics and Modelling.

[CR20] Dunn WB (2014). Molecular phenotyping of a UK population: Defining the human serum metabolome. Metabolomics.

[CR21] Durant JL, Leland BA, Henry DR, Nourse JG (2002). Reoptimization of MDL keys for use in drug discovery. Journal of Chemical Information and Computer Sciences.

[CR22] Eckert H, Bajorath J (2007). Molecular similarity analysis in virtual screening: Foundations, limitations and novel approaches. Drug Discovery Today.

[CR23] Eisen MB, Spellman PT, Brown PO, Botstein D (1998). Cluster analysis and display of genome-wide expression patterns. Proceedings of National Academy of Sciences.

[CR24] Empfield JR, Leeson PD (2010). Lessons learned from candidate drug attrition. IDrugs.

[CR25] Everitt BS (1993). Cluster analysis.

[CR26] Faulon J-L, Bender A (2010). Handbook of chemoinformatics algorithms.

[CR27] Feher M, Schmidt JM (2003). Property distributions: Differences between drugs, natural products, and molecules from combinatorial chemistry. Journal of Chemical Information and Computer Sciences.

[CR28] Flower DR (1998). On the properties of bit string-based measures of chemical similarity. Journal of Chemical Information and Computer Sciences.

[CR29] Gamo FJ (2010). Thousands of chemical starting points for antimalarial lead identification. Nature.

[CR30] Gasteiger J (2003). Handbook of Chemoinformatics: From data to knowledge.

[CR31] Giacomini KM, Huang SM (2013). Transporters in drug development and clinical pharmacology. Clinical Pharmacology and Therapeutics.

[CR32] Giacomini KM (2010). Membrane transporters in drug development. Nature Reviews Drug Discovery.

[CR33] Gozalbes R, Pineda-Lucena A (2011). Small molecule databases and chemical descriptors useful in chemoinformatics: An overview. Combinatorial Chemistry & High Throughput Screening.

[CR34] Guiguemde WA (2010). Chemical genetics of *Plasmodium falciparum*. Nature.

[CR35] Gupta S, Aires-de-Sousa J (2007). Comparing the chemical spaces of metabolites and available chemicals: Models of metabolite-likeness. Molecular Diversity.

[CR36] Hamdalla MA, Mandoiu, Hill DW, Rajasekaran S, Grant DF (2013). BioSM: Metabolomics tool for identifying endogenous mammalian biochemical structures in chemical structure space. Journal of Chemical Information and Modeling.

[CR37] Handl J, Knowles J (2007). An evolutionary approach to multiobjective clustering. IEEE Transactions on Evolutionary Computation.

[CR38] Handl J, Knowles J, Kell DB (2005). Computational cluster validation in post-genomic data analysis. Bioinformatics.

[CR39] Hastings J (2013). The ChEBI reference database and ontology for biologically relevant chemistry: Enhancements for 2013. Nucleic Acids Research.

[CR40] Haug K (2013). MetaboLights-an open-access general-purpose repository for metabolomics studies and associated meta-data. Nucleic Acids Research.

[CR41] Hay M, Thomas DW, Craighead JL, Economides C, Rosenthal J (2014). Clinical development success rates for investigational drugs. Nature Biotechnology.

[CR42] Herrgård MJ (2008). A consensus yeast metabolic network obtained from a community approach to systems biology. Nature Biotechnology.

[CR43] Hert J, Irwin JJ, Laggner C, Keiser MJ, Shoichet BK (2009). Quantifying biogenic bias in screening libraries. Nature Chemical Biology.

[CR44] Holdgate GA (2007). Thermodynamics of binding interactions in the rational drug design process. Expert Opinion on Drug Discovery.

[CR45] Hopkins AL, Keserü GM, Leeson PD, Rees DC, Reynolds CH (2014). The role of ligand efficiency metrics in drug discovery. Nature Reviews Drug Discovery.

[CR46] Huttunen KM, Raunio H, Rautio J (2011). Prodrugs–from serendipity to rational design. Pharmacological Reviews.

[CR47] Irwin JJ, Sterling T, Mysinger MM, Bolstad ES, Coleman RG (2012). ZINC: A free tool to discover chemistry for biology. Journal of Chemical Information and Modeling.

[CR48] Kanehisa M, Goto S, Sato Y, Furumichi M, Tanabe M (2012). KEGG for integration and interpretation of large-scale molecular data sets. Nucleic Acids Research.

[CR49] Kanehisa M, Goto S, Sato Y, Kawashima M, Furumichi M, Tanabe M (2014). Data, information, knowledge and principle: Back to metabolism in KEGG. Nucleic Acids Research.

[CR50] Karakoc E, Sahinalp SC, Cherkasov A (2006). Comparative QSAR- and fragments distribution analysis of drugs, druglikes, metabolic substances, and antimicrobial compounds. Journal of Chemical Information and Modeling.

[CR51] Karp PD, Caspi R (2011). A survey of metabolic databases emphasizing the MetaCyc family. Archives of Toxicology.

[CR52] Kell DB (2012). Scientific discovery as a combinatorial optimisation problem: How best to navigate the landscape of possible experiments?. BioEssays.

[CR53] Kell DB (2013). Finding novel pharmaceuticals in the systems biology era using multiple effective drug targets, phenotypic screening, and knowledge of transporters: Where drug discovery went wrong and how to fix it. FEBS Journal.

[CR54] Kell, D. B., Dobson, P. D. (2009). The cellular uptake of pharmaceutical drugs is mainly carrier-mediated and is thus an issue not so much of biophysics but of systems biology. In M. G. Hicks, & C. Kettner (Eds.), *Proceedings of International Beilstein Symposium on Systems Chemistry* (pp. 149–168). Berlin: Logos. http://www.beilstein-institut.de/Bozen2008/Proceedings/Kell/Kell.pdf.

[CR55] Kell DB, Dobson PD, Bilsland E, Oliver SG (2013). The promiscuous binding of pharmaceutical drugs and their transporter-mediated uptake into cells: What we (need to) know and how we can do so. Drug Discovery Today.

[CR56] Kell DB, Dobson PD, Oliver SG (2011). Pharmaceutical drug transport: The issues and the implications that it is essentially carrier-mediated only. Drug Discovery Today.

[CR57] Kell DB, Goodacre R (2014). Metabolomics and systems pharmacology: Why and how to model the human metabolic network for drug discovery. Drug Discovery Today.

[CR58] Khanna V, Ranganathan S (2009). Physicochemical property space distribution among human metabolites, drugs and toxins. BMC Bioinformatics.

[CR59] Khanna V, Ranganathan S (2011). Structural diversity of biologically interesting datasets: A scaffold analysis approach. Journal of Cheminformatics.

[CR60] Knight CG (2009). Array-based evolution of DNA aptamers allows modelling of an explicit sequence-fitness landscape. Nucleic Acids Research.

[CR61] Knox C (2011). DrugBank 3.0: A comprehensive resource for ‘omics’ research on drugs. Nucleic Acids Research.

[CR62] Kola I (2008). The state of innovation in drug development. Clinical Pharmacology and Therapeutics.

[CR63] Kola I, Landis J (2004). Can the pharmaceutical industry reduce attrition rates?. Nature Reviews Drug Discovery.

[CR64] Koutsoukas, A., et al. (2013). How diverse are diversity assessment methods? A comparative analysis and benchmarking of molecular descriptor space. *Journal of Chemical Information and Modeling*. doi:10.1021/ci400469u. 10.1021/ci400469u 24289493

[CR65] Landrum G, Lewis R, Palmer A, Stiefl N, Vulpetti A (2011). Making sure there’s a “give” associated with the “take”: Producing and using open-source software in big pharma. Journal of Cheminformatics.

[CR66] Lanthaler K (2011). Genome-wide assessment of the carriers involved in the cellular uptake of drugs: A model system in yeast. BMC Biology.

[CR67] Law V (2014). DrugBank 4.0: Shedding new light on drug metabolism. Nucleic Acids Research.

[CR68] Li P, Oinn T, Soiland S, Kell DB (2008). Automated manipulation of systems biology models using libSBML within Taverna workflows. Bioinformatics.

[CR69] Li P (2008). Performing statistical analyses on quantitative data in Taverna workflows: An example using R and maxdBrowse to identify differentially expressed genes from microarray data. BMC Bioinformatics.

[CR70] Lipinski CA, Lombardo F, Dominy BW, Feeney PJ (1997). Experimental and computational approaches to estimate solubility and permeability in drug discovery and development settings. Advanced Drug Delivery Reviews.

[CR71] Lucas X (2013). StreptomeDB: A resource for natural compounds isolated from *Streptomyces* species. Nucleic Acids Research.

[CR72] Maggiora G, Vogt M, Stumpfe D, Bajorath J (2014). Molecular similarity in medicinal chemistry. Journal of Medicinal Chemistry.

[CR73] Maldonado AG, Doucet JP, Petitjean M, Fan BT (2006). Molecular similarity and diversity in chemoinformatics: From theory to applications. Molecular Diversity.

[CR74] Mazanetz MP, Marmon RJ, Reisser CBT, Morao I (2012). Drug discovery applications for KNIME: An open source data mining platform. Current Topics in Medicinal Chemistry.

[CR75] McGregor MJ, Pallai PV (1997). Clustering of large databases of compounds: Using the MDL ‘‘keys’’ as structural descriptors. Journal of Chemical Information and Computer Sciences.

[CR76] Medina-Franco JL, Maggiora GM, Bajorath J (2014). Molecular similarity analysis. Chemoinformatics for drug discovery.

[CR77] Meinl, T., Jagla, B., Berthold, M. R. (2012). Integrated data analysis with KNIME. Open source software in life science research: Practical solutions in the pharmaceutical industry and beyond, pp. 151–171. doi:10.1533/9781908818249.

[CR78] Muchmore SW, Debe DA, Metz JT, Brown SP, Martin YC, Hajduk PJ (2008). Application of belief theory to similarity data fusion for use in analog searching and lead hopping. Journal of Chemical Information and Modeling.

[CR79] Newman DJ, Cragg GM (2012). Natural products as sources of new drugs over the 30 years from 1981 to 2010. Journal of Natural Products.

[CR80] Ohno K, Nagahara Y, Tsunoyama K, Orita M (2010). Are there differences between launched drugs, clinical candidates, and commercially available compounds?. Journal of Chemical Information and Modeling.

[CR81] Ooi HS, Schneider G, Lim TT, Chan YL, Eisenhaber B, Eisenhaber F (2010). Biomolecular pathway databases. Methods and Molecular Biology.

[CR82] Oprea TI (2004). Chemoinformatics in drug discovery.

[CR83] Oprea TI, Allu TK, Fara DC, Rad RF, Ostopovici L, Bologa CG (2007). Lead-like, drug-like or “Pub-like”: How different are they?. Journal of Computer-Aided Molecular Design.

[CR84] Oprea TI, Davis AM, Teague SJ, Leeson PD (2001). Is there a difference between leads and drugs? A historical perspective. Journal of Chemical Information and Computer Sciences.

[CR85] Overington JP, Al-Lazikani B, Hopkins AL (2006). How many drug targets are there?. Nature Reviews Drug Discovery.

[CR86] Paolini GV, Shapland RH, van Hoorn WP, Mason JS, Hopkins AL (2006). Global mapping of pharmacological space. Nature Biotechnology.

[CR87] Papadatos G (2010). Lead optimization using matched molecular pairs: Inclusion of contextual information for enhanced prediction of hERG inhibition, solubility, and lipophilicity. Journal of Chemical Information and Modeling.

[CR88] Peironcely JE, Reijmers T, Coulier L, Bender A, Hankemeier T (2011). Understanding and classifying metabolite space and metabolite-likeness. PLoS One.

[CR89] Rafols I (2014). Big Pharma, little science? A bibliometric perspective on Big Pharma’s R&D decline. Technological Forecasting and Social Change.

[CR90] Riniker S, Landrum GA (2013). Open-source platform to benchmark fingerprints for ligand-based virtual screening. Journal of Cheminformatics.

[CR91] Riniker S, Landrum GA (2013). Similarity maps—A visualization strategy for molecular fingerprints and machine-learning methods. Journal of Cheminformatics.

[CR92] Sastry M, Lowrie JF, Dixon SL, Sherman W (2010). Large-scale systematic analysis of 2D fingerprint methods and parameters to improve virtual screening enrichments. Journal of Chemical Information and Modeling.

[CR93] Saubern S, Guha R, Baell JB (2011). KNIME workflow to assess PAINS filters in SMARTS format. Comparison of RDKit and Indigo Cheminformatics Libraries. Molecular Informatics.

[CR94] Sheridan RP, Feuston BP, Maiorov VN, Kearsley SK (2004). Similarity to molecules in the training set is a good discriminator for prediction accuracy in QSAR. Journal of Chemical Information and Computer Sciences.

[CR95] Sheridan RP, Kearsley SK (2002). Why do we need so many chemical similarity search methods?. Drug Discovery Today.

[CR96] Small BG (2011). Efficient discovery of anti-inflammatory small molecule combinations using evolutionary computing. Nature Chemical Biology.

[CR97] Steinbeck C, Han YQ, Kuhn S, Horlacher O, Luttmann E, Willighagen E (2003). The Chemistry Development Kit (CDK): An open-source Java library for chemo- and bioinformatics. Journal of Chemical Information and Computer Sciences.

[CR98] Stöter M, Niederlein A, Barsacchi R, Meyenhofer F, Brandl H, Bickle M (2013). Cell Profiler and KNIME: Open source tools for high content screening. Methods and Molecular Biology.

[CR99] Swainston N, Mendes P (2009). libAnnotationSBML: A library for exploiting SBML annotations. Bioinformatics.

[CR100] Swainston N, Mendes P, Kell DB (2013). An analysis of a ‘community-driven’ reconstruction of the human metabolic network. Metabolomics.

[CR101] Thiele I (2013). A community-driven global reconstruction of human metabolism. Nature Biotechnology.

[CR102] Todeschini R, Consonni V (2000). Handbook of molecular descriptors.

[CR103] van der Greef J, McBurney RN (2005). Rescuing drug discovery: In vivo systems pathology and systems pharmacology. Nature Reviews Drug Discovery.

[CR104] van Deursen R, Blum LC, Reymond JL (2011). Visualisation of the chemical space of fragments, lead-like and drug-like molecules in PubChem. Journal of Computer-Aided Molecular Design.

[CR105] Walters WP (2012). Going further than Lipinski’s rule in drug design. Expert Opinion on Drug Discovery.

[CR106] Walters WP, Green J, Weiss JR, Murcko MA (2011). What do medicinal chemists actually make? A 50-year retrospective. Journal of Medicinal Chemistry.

[CR107] Wang Y, Bajorath J (2010). Advanced fingerprint methods for similarity searching: Balancing molecular complexity effects. Combinatorial Chemistry & High Throughput Screen.

[CR108] Warr WA (2012). Scientific workflow systems: Pipeline Pilot and KNIME. Journal of Computer-Aided Molecular Design.

[CR109] Willett P (2006). Similarity-based virtual screening using 2D fingerprints. Drug Discovery Today.

[CR110] Wishart DS (2013). HMDB 3.0—The Human Metabolome Database in 2013. Nucleic Acids Research.

[CR111] Wunberg T (2006). Improving the hit-to-lead process: Data-driven assessment of drug-like and lead-like screening hits. Drug Discovery Today.

[CR112] Zhang MQ, Wilkinson B (2007). Drug discovery beyond the ‘rule-of-five’. Current Opinion in Biotechnology.

[CR113] Zhang J, Lushington GH, Huan J (2011). Characterizing the diversity and biological relevance of the MLPCN assay manifold and screening set. Journal of Chemical Information and Modeling.

